# Generic catastrophic poverty when selfish investors exploit a degradable common resource

**DOI:** 10.1098/rsos.221234

**Published:** 2023-02-08

**Authors:** Claudius Gros

**Affiliations:** Institute for Theoretical Physics, Goethe University Frankfurt, Frankfurt, Germany

**Keywords:** game theory, resource exploitation, tragedy of the commons, catastrophic poverty

## Abstract

The productivity of a common pool of resources may degrade when overly exploited by a number of selfish investors, a situation known as the tragedy of the commons. Without regulations, agents optimize the size of their individual investments into the commons by balancing incurring costs with the returns received. The resulting Nash equilibrium involves a self-consistency loop between individual investment decisions and the state of the commons. As a consequence, several non-trivial properties emerge. For *N* investing actors we prove rigorously that typical payoffs do not scale as 1/*N*, the expected result for cooperating agents, but as (1/*N*)^2^. Payoffs are hence reduced with regard to the functional dependence on *N*, a situation denoted catastrophic poverty. We show that catastrophic poverty results from a fine-tuned balance between returns and costs. Additionally, a finite number of oligarchs may be present. Oligarchs are characterized by payoffs that are finite and not decreasing when *N* increases. Our results hold for generic classes of models, including convex and moderately concave cost functions. For strongly concave cost functions the Nash equilibrium undergoes a collective reorganization, being characterized instead by entry barriers and sudden death forced market exits.

## 1. Introduction

The tragedy of the commons (TOC) [1] occurs when a common resource, the commons, is overly exploited by a number of selfish agents [2]. Unmanaged access to the commons allows for the maximization of individual profits, which may lead in turn to a potentially severe reduction of overall welfare. Given the essential importance of land, water and other environmental resources, it may not surprise that human societies developed over time a large array of regulative options for their preservation [3,4]. On a global level it is, however, not yet clear whether humanity’s present course of actions will, or will not, lead to a world-wide TOC [5,6]. Over-exploitation can be avoided from a game-theoretical perspective when appropriate rules are introduced [7]. In this context, the role of evolving strategies [8,9] has been discussed, as well as situations that can be modelled using cooperation or coordination games [6,10]. Analogous questions arise with regard to the interplay between the utilization of common resources and Darwinian competition within natural habitats [11–13]. On a social level, conflicting objectives between individual and group interests are ubiquitous [14], for instance when it comes to coping with climate changes [15], and in the context of vaccination campaigns [16,17]. With regards to the time domain, it has been shown that the dynamics of the feedback loop between environmental degradation and individual investment decisions may lead to a large variety of complex dynamical states [18–20].

A wide range of situations involve the TOC [21]. In a basic framework, a group of agents invests independently into a common resource [22]. The returns received are, however, dependent on the status of the commons, which degrades as a function of overall investment. A reference example is the depletion of an extended underground aquifer when the distributed extraction of water remains unchecked. Without coordination, pumping continues as long as it is economically viable. The size of the profit made is not directly relevant in this situation, as long as it remains positive. Falling levels of the water reservoir will require, however, deeper and more powerful wells, which is equivalent to a decreasing productivity of the commons.

Here, we will not investigate how the TOC may be averted. Instead, we examine in detail the TOC equilibrium, namely the steady state resulting from individual profit maximization. In this state, the degree of exploitation remains finite for all numbers of participating agents, becoming potentially large only for small investment costs. Intuitively one may expect that agents receive individual payoffs of the order of 1/*N*, where *N* is the number of participating agents. This is, however, not the case. We prove that payoffs scale as (1/*N*)^2^ for the majority of agents. A scaling with (1/*N*)^2^ entails that payoffs are dramatically reduced when *N* is large, a situation denoted ‘catastrophic poverty’. Using numerical simulations, we find that this effect becomes relevant already for *N* ∼10–20. In addition to the agents suffering from catastrophic poverty, there may exist a finite number of investors with substantial profits, the oligarchs. In the TOC equilibrium, the group of oligarchs is finite in the sense that their number cannot be proportional to *N*.

An analogy can be made to the optimization of production in an elastic market [23]. Firms produce goods at a factor price, the per-unit cost, for which they receive a market price that decreases when the total number of goods produced is increased. Market prices are in this analogy identical for all producers, but not the individual cost functions. We prove that profits are quadratic and not linear functions of per-unit costs when firms optimize their outputs individually. This observation holds in analogy for the TOC equilibrium.

In the first step, a basic reference model is used for the development of the framework. Subsequently, we show that catastrophic poverty arises generically when marginal costs are either constant, increasing or moderately decreasing when the investment into the commons is increased. The nature of the Nash equilibrium changes, however, qualitatively when economies of scale become pronounced, which corresponds to the case of strongly concave cost functions. In this regime catastrophic poverty is absent. Instead, new investors with higher costs will face a barrier to market entry. The entry barrier arises because equilibrium investment levels are governed by a saddle–node bifurcation, which changes also the timescale of forced market exits.

## 2. Reference model for the tragedy of the commons

In a first step, we use a basic reference model for the development of the discussion. We will keep the model as simple as possible, showing subsequently that the conclusions reached hold for frameworks generalized along several directions.

**Figure 1.**
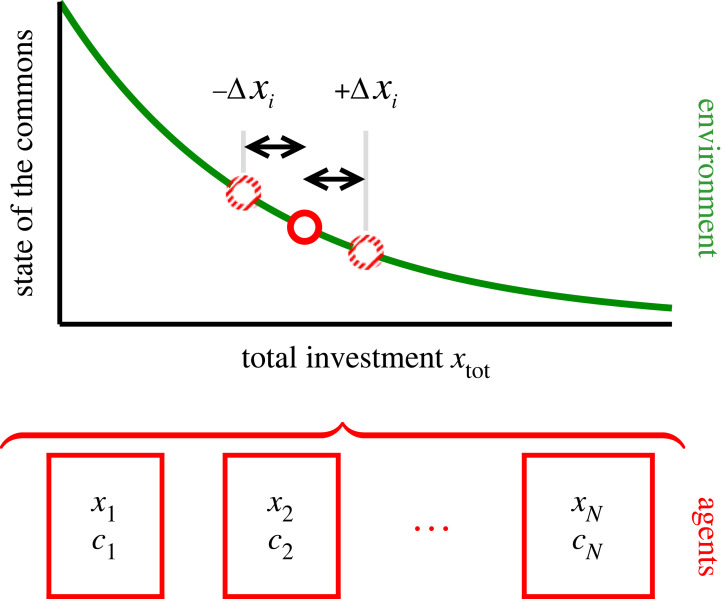
Modelling framework. Agents contribute *x*_*i*_ to the total investment $x_{\mathrm{tot}}=\sum_{i}x_{i}$, which determines in turn the degree of exploitation of the commons (‘the state’). Productivity (green line) decreases with rising levels of exploitation, reacting in response to a change by ±Δ*x*_*i*_ of an individual contribution. Payoffs per investment are given by the productivity of the commons minus the individual per-unit costs *c*_*i*_.

Agents receive payoffs *E*_*i*_ from investing amounts *x*_*i*_ ≥ 0 into the commons. Here *i* = 1, …, *N*, with *N* being the number of participating agents. Investments of all sizes are allowed as long as payoffs remain positive. The payoffs are given by the difference between the nominal return and investment costs,

2.1 $$E_{i}=(e^{-x_{\mathrm{tot}}}-c_{i})x_{i}\;\text{and}\;x_{\mathrm{tot}}=\sum_{j}x_{j},$$

where *c*_*i*_ > 0 is the per-unit cost of the agent. Marginal costs are assumed here to be constant, a specification that will be relaxed further on. The factor exp (− *x*_tot_) specifies how the productivity of the commons (the nominal return per unit investment) decreases as a function of total investments, *x*_tot_. Alternative functional forms for the decay of the productivity with increasing exploitation will be discussed further down together with a close relation of (2.1) to an experimental protocol used by Ostrom [22]. A graphical illustration of our approach is given in figure 1.

### 2.1. Finite productivity

Formally, the common pool of resources defined with (2.1) has an infinite size, in the sense that *x*_*i*_ can be arbitrarily large. The effective productivity of the commons is nevertheless limited. This is evident when considering the case of a single investor, *N* = 1, setting *x* = *x*_*i*_ and *c* = *c*_*i*_. The payoff is

2.2 $$E=x(e^{-x}-c),$$

which becomes negative when exp (− *x*) < *c*. Optimality is reached when d*E*/d*x* = 0, which leads to

2.3 $$(1-x)e^{-x}=c\;\text{and}\;E{|}_{\mathrm{opt}}=x^{2}e^{-x}.$$

**Figure 2.**
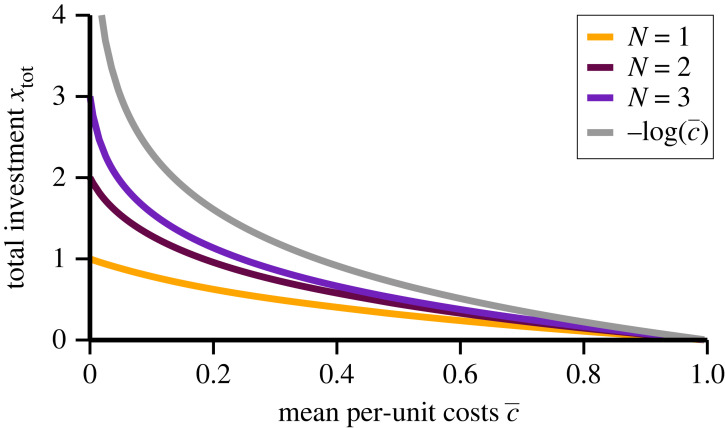
Optimal total investments. For various numbers of agents, *N*, the solution of the self-consistency condition for *x*_tot_, equation (2.5). The data interpolate smoothly between *x*_tot_ = *N* at $\bar{c}=0$ and *x*_tot_ = 0 for $\bar{c}=1$. For *N* → ∞ one has $x_{\mathrm{tot}}=\log(1/\bar{c})$, which diverges logarithmically for vanishing average costs, $\bar{c}\to 0$.

The solution of the self-consistency condition for *x*, the first equation in (2.3), leads to a finite optimal investment, 0 ≤ *x* ≤ 1, which interpolates smoothly between *x* = 0 (for *c* = 1) and *x* = 1 (for *c* = 0), as shown in figure 2. The decaying productivity of the commons leads to optimal investments that are finite even when investment costs vanish, *viz.* when *c* → 0. Later on we will show that it does not matter whether the size of the commons is formally infinite, as defined in (2.1), or finite from the start.

### 2.2. Selfish individuals

Selfish agents increase their investments *x*_*i*_ until the individual gradients d*E*_*i*_/d*x*_*i*_ vanish. This leads to

2.4 $$(1-x_{i})e^{-x_{\mathrm{tot}}}=c_{i}\;\text{and}\;x_{i}=1-c_{i}e^{x_{\mathrm{tot}}},$$

in analogy to the case of an individual investor discussed above. Here, we used that d*x*_tot_/d*x*_*i*_ = 1. In the stationary state, individual investments are strictly linear in the *c*_*i*_. Equation (2.4) describes *N* self-consistency conditions for *N* variables, the individual *x*_*i*_, which are coupled through *x*_tot_.^1^ The size of the total investment *x*_tot_ can be determined by averaging (2.4) over all agents,

2.5 $$\left(1-\frac{x_{\mathrm{tot}}}{N}\right)e^{-x_{\mathrm{tot}}}=\bar{c}\;\text{and}\;\bar{c}=\frac{1}{N}\sum_{i}c_{i},$$

where $\bar{c}$ is the mean per unit cost. The cumulative investment is hence solely a function of *N* and of the average costs. In particular, it does not depend on the actual distribution of the respective *c*_*i*_. A finite solution exists for all $0<\bar{c}<1$, as illustrated in figure 2. In the large-*N* limit one finds $x_{\mathrm{tot}}\to \log(1/\bar{c})$.

### 2.3. Initial decimation

The investment *x*_*i*_ resulting from self-centred optimization is

2.6 $$x_{i}=1-\frac{c_{i}}{c_{\max}}\;\text{and}\;c_{\max}=e^{-x_{\mathrm{tot}}}$$

which follows from (2.4). Per-unit costs exceeding *c*_max_ lead to *x*_*i*_ < 0, which would result in negative payoffs. When this happens, the agent in question is assumed to quit the market. Otherwise, agents continue to exploit the common resource independently of the absolute size of the profit made. It is assumed that it does not matter for the individual whether other agents receive smaller or larger payoffs, as long as profits can be made. In the framework examined here, direct competition between agents is absent.

The Nash equilibrium depends via (2.5) on $\bar{c}$ and *N*, which are both reduced when an agent with negative payoffs drops out of the market. This will lead to a reduced profitability limit *c*_max_ = exp (−*x*_tot_), with the consequence that additional agents may become unprofitable. This iterated process is denoted ‘decimation’.

For the initial decimation process, one starts with a given distribution for the per-unit costs *c*_*i*_. One then calculates $\bar{c}$ and *x*_tot_, the latter from (2.5). Next *c*_max_ is determined and agents with *c*_*i*_ > *c*_max_ eliminated. One starts over again with renormalized quantities:

2.7 $$N=\sum_{i,E_{i}>0}1\;\text{and}\;\bar{c}=\frac{1}{N}\sum_{i,E_{i}>0}c_{i}.$$

This procedure is iterated until convergence. From now on only the population surviving the initial decimation process is considered, with *N* and $\bar{c}$ corresponding, respectively, to the number and to the average per-unit costs of the surviving agents.

### 2.4. Dispersion relation

Combining (2.6) and (2.1) results in

2.8 $$E_{i}{|}_{\mathrm{opt}}=x_{i}(e^{-x_{\mathrm{tot}}}-c_{i}){|}_{\mathrm{opt}}=\left(1-\frac{c_{i}}{c_{\max}}\right)(c_{\max}-c_{i}),$$

or

2.9 $$E(c_{i})\equiv E_{i}{|}_{\mathrm{opt}}=c_{\max}{\left(1-\frac{c_{i}}{c_{\max}}\right)}^{2}.$$

This expression holds for the stationary state, *viz.* for the Nash equilibrium. It is universal in the sense that it depends on $\bar{c}$ and *N* only implicitly via *c*_max_, but not explicitly.

Equation (2.9) has the form of a dispersion relation, a notation borrowed from physics,^2^ providing a distinct functional relation between *c*_*i*_ and the respective payoff. It is quite remarkable that the dispersion is strictly quadratic over the entire range, *c*_*i*_ ∈ [0, *c*_max_], and not only close to marginal profitability. In the latter regime, *viz.* when *c*_*i*_ is close to *c*_max_, the quadratic dependency arises because the optimal investment *x*_*i*_ = 1 − *c*_*i*_/*c*_max_ vanishes linearly when *c*_*i*_ → *c*_max_.

### 2.5. Catastrophic poverty

It follows from (2.5) that

2.10 $$\frac{\bar{c}}{c_{\max}}=\frac{\bar{c}}{e^{-x_{\mathrm{tot}}}}=1-\frac{x_{\mathrm{tot}}}{N}\approx 1-\frac{\log(1/\bar{c})}{N},$$

where we used in the last step the large-*N* expression $x_{\mathrm{tot}}\to \log(1/\bar{c})$ for the overall investment. The relative distance of the profitability limit *c*_max_ to the mean per-unit costs $\bar{c}$ vanishes hence in the large *N* limit when $\bar{c}>0$. This has dramatic consequences for the size of the payoffs of less competitive investors.

Inserting (2.10) into the dispersion relation, *E*_*i*_|_opt_ = *c*_max_(1 − *c*_*i*_/*c*_max_)^2^, yields

2.11 $$E_{i}{|}_{\mathrm{opt}}\;\overset{c_{i}=\bar{c}}{--⟶}\;\frac{\bar{c}}{1-x_{\mathrm{tot}}/N}{\left(\frac{x_{\mathrm{tot}}}{N}\right)}^{2}∼\frac{1}{N^{2}}$$

for the payoff of an average agents, defined by $c_{i}=\bar{c}$. We assumed for the last step that *x*_tot_ is not diverging with *N*, which is the case when one considers a sequence of populations with increasing sizes, but fixed average per-unit costs $\bar{c}$. The result is, somewhat surprisingly, that the optimal individual payoff scales with (1/*N*)^2^.

Equation (2.11) is a non-trivial result. A finite cumulative payoff $E_{\mathrm{tot}}=\sum_{i}E_{i}$ can be extracted from the common resource and one could have expected that everybody gets a share scaling with 1/*N*, with the actual size being a function of individual per-unit costs *c*_*i*_. This is, however, not the case. The balance between costs and returns becomes increasingly fine-tuned with larger and large *N*, which drives *c*_max_ successively closer to $\bar{c}$.

Payoffs drop continuously with increasing per-unit costs, *c*_*i*_, which means that the (1/*N*)^2^ scaling following from (2.11) holds at least for all $\bar{c}\leq c_{i}<c_{\max}$. In general, there is a finite fraction of agents in the interval $\bar{c}\leq c_{i}<c_{\max}$, typically about half the population is situated in this region. The payoffs of these agents are not just small, but functionally depressed, in the sense that payoff reduction is due to a specific functional dependency on *N*. This situation is denoted here as ‘catastrophic poverty’.

The origins of catastrophic poverty can be traced to three properties of the dispersion relation *E*(*c*_*i*_):

— *c*_max_. An upper bound for per-unit costs exists. Investments become non-profitable above, with *E*(*c*_max_) = 0.
— $c_{\max}-\bar{c}∼1/N$. The mean per-unit costs approach the upper bound when the number of agents increases. Compare (2.10).
— *E*(*c*_*i*_) ∼ (*c*_max_ − *c*_*i*_)^2^. A quadratic dispersion relation implies that payoffs vanish quadratically close to *c*_max_.

Any Nash state with a dispersion relation fulfilling these three properties is characterized by catastrophic poverty. We will show later on that this is the case not only for the basic model specified by equation (2.1), but for a wide class of generic frameworks describing the exploitation of a common resource by selfish agents.

### 2.6. Wealthy oligarchs

Catastrophic poverty affects a substantial part of the population, but not necessarily all agents. A few individuals, the oligarchs, may have substantial payoffs. Whether oligarchs exist depends on the distribution {*c*_*i*_} of the per-unit costs of the surviving agents.

We start with two illustrative examples for the cost-distribution {*c*_*i*_}. In the first case, the per-unit costs of the *N*-agents are uniformly distributed around the mean, in the interval $\left[\bar{c}-\delta c,\bar{c}+\delta c\right]$, where $\delta c<c_{\max}-\bar{c}$. The distance to the boundary, *c*_max_ − *c*_*i*_, scales, therefore, as 1/*N* for all agents. Given that the dispersion relation *E*(*c*_*i*_) is quadratic, one has *E*(*c*_*i*_) ∼ (1/*N*)^2^ for all agents. In this example, catastrophic poverty affects the entire population.

In the second example, a single agent has vanishing production costs, *c*_*i*_ = 0, with the other agents having the identical *c*_*i*_, located at $\bar{c}+\delta c$, with $\delta c=\alpha (c_{\max}-\bar{c})$ and *α* < 1. The average is reproduced for

2.12 $$\bar{c}=\frac{N-1}{N}[\bar{c}+\alpha (c_{\max}-\bar{c})]\;\text{and}\;\alpha =\frac{1}{N-1}\frac{\bar{c}}{c_{\max}-\bar{c}}.$$

Using (2.10) leads to

2.13 $$\alpha =\frac{N}{N-1}\frac{1}{x_{\mathrm{tot}}}\approx \frac{1}{x_{\mathrm{tot}}}.$$

The profitability condition *α* < 1 is satisfied for *x*_tot_ > 1, which holds for a large range of $\bar{c}$ and *N*, as evident from the data presented in figure 2. The payoff of the oligarch is

2.14 $$E(0)=c_{\max}=\frac{\bar{c}}{1-x_{\mathrm{tot}}/N},$$

which is finite whenever $\bar{c}>0$. Note that (2.10) has been used for *c*_max_ and that (2.14) holds for *N* that are large enough for (2.13) to be fulfilled. As a consequence, the profit made by the oligarch can be orders of magnitudes larger than the profit *E*(*c*_*i*_) = (*c*_max_ − *c*_*i*_)^2^/*c*_max_ of any of the remaining *N* − 1 agents,

2.15 $$E(c_{i})=\frac{{\left(x_{\mathrm{tot}}-1\right)}^{2}}{x_{\mathrm{tot}}^{2}}\frac{{\left(c_{\max}-\bar{c}\right)}^{2}}{c_{\max}}\;\text{and}\;c_{i}=\bar{c}+\frac{c_{\max}-\bar{c}}{x_{\mathrm{tot}}},$$

which are all suffering from catastrophic poverty when the size of the population is substantial.

When oligarchs have finite payoffs, as in the above example, the cumulative payoff is also finite. For the general case, we reorganize the dispersion relation (2.9) as

2.16 $$E_{i}(c_{i})=c_{\max}-2c_{i}+\frac{c_{i}^{2}-{\bar{c}}^{2}+{\bar{c}}^{2}}{c_{\max}},$$

where we added and subtracted ${\bar{c}}^{2}$ in the numerator of the last term. Taking the average over all agents yields $\bar{E}=\sum_{i}E_{i}(c_{i})/N$,

2.17 $$\bar{E}=\frac{{\left(c_{\max}-\bar{c}\right)}^{2}}{c_{\max}}+\frac{\sigma_{c}^{2}}{c_{\max}},$$

where $\sigma_{c}^{2}=\sum_{i}(c_{i}^{2}-{\bar{c}}^{2})/N$ is the variance of the per-unit costs. The first term in (2.17) scales as (1/*N*)^2^, as shown previously, see (2.10). The overall scaling of $\bar{E}$ depends, therefore, on the scaling of the variance $\sigma_{c}^{2}$ with *N*.

For the first example discussed above, we have *σ*_*c*_ ∼ 1/*N*. Consistently, the average payoff is proportional to (1/*N*)^2^. The cumulative payoff $N\bar{E}$ scales, therefore, as 1/*N*, vanishing in the limit of large populations. Everybody suffers.

In our second example, an oligarch with a finite payoff is present, which implies a finite cumulative payoff $N\bar{E}$ and that $\bar{E}$ scales as 1/*N*. This is consistent with the scaling of the variance $\sigma_{c}^{2}$, to which the per-unit cost *c*_*i*_ = 0 of the oligarch contributes a term ${\bar{c}}^{2}/N$. The upshot is that the presence of one or more oligarchs increases average payoffs, however, without eliminating catastrophic poverty for the majority of agents.

Conceptually, our approach is based on the analysis of a series of distributions {*c*_*i*_} for the player specific per-unit costs, with the premise that these distributions can be defined consistently for various population sizes *N*. In practice, the final distribution {*c*_*i*_} of the surviving agents results from the initial decimation process. Performing numerical simulations, as described further below in more detail, we found that the two types of Nash-states discussed here appear readily from decimation for a wide range of starting allocations for the agent-specific cost structure.

### 2.7. Cooperation

For the analysis performed so far we assumed that agents do not cooperate. For comparison we investigate now the possible benefits of coordinating investments. Our purpose is, however, not to develop a generic theory of cooperation, for which there would be a range of distinct optimization principles. Cooperation is uniquely defined only when agents are identical, *viz.* when *c*_*i*_ ≡ *c*. In general, an objective could be to optimize the mean payoff $\bar{E}$, either alone or in conjunction with a given criterion for the fairness of income distributions, like the Gini index. An additional variable is the number of surviving agents. It could be desirable to keep the size of the population as large as possible when investing cooperatively, or not. Here, we restrict ourselves to a basic discussion.

As a reference protocol we consider that agents invest equal shares, *x*_*i*_ ≡ *x*_tot_/*N*, which is optimal for the case of identical agents. The aim is then to optimize total payoff, $E_{\mathrm{tot}}=\sum_{i}E_{i}$, namely

2.18 $$E_{\mathrm{tot}}=(c_{\max}-\bar{c})x_{\mathrm{tot}}\;\text{and}\;c_{\max}=e^{-x_{\mathrm{tot}}}.$$

In effect, the community of agents acts as a single investor, see (2.2) and (2.3). The consequence is that the cumulative payoff is finite when agents cooperate. For the individual agent we have

2.19 $$E_{i}{|}_{\mathrm{coop}}=(c_{\max}-c_{i})\frac{x_{\mathrm{tot}}}{N},$$

which scales as 1/*N*. Catastrophic poverty is avoided when cooperating, with each surviving agent receiving a fair share. This is consistent with the result that the dispersion relation is linear, as expressed by (2.19), which corresponds to the classical expectation. The overall level of exploitation remains comparatively low, as *x*_tot_ < 1 for *N* = 1, as shown in figure 2. If follows from *c*_max_ = exp (− *x*_tot_) that *c*_max_ < 1. High-cost agents will, therefore, be driven out of the market during the initial decimation process even when everybody cooperates according to the protocol considered here. By contrast, decimation will have no effect when agents have identical investment costs.

### 2.8. Generic productivity function

Catastrophic poverty arises in large classes of models describing the exploitation of a common resource by non-cooperating agents. In the basic model (2.1), the commons degraded exponentially with total investments, as exp (− *x*_tot_). On a general level we denote with *P*(*x*_tot_) the productivity of the commons. The respective payoff function is then

2.20 $$E_{i}=x_{i}[P(x_{\mathrm{tot}})-c_{i}]\;\text{with}\;P(0)=1\;\text{and}\;P^{\prime }(x_{\mathrm{tot}})<0.$$

Payoffs are optimal for

2.21 $$P(x_{\mathrm{tot}})-c_{i}=-x_{i}P^{\prime }(x_{\mathrm{tot}})\;\text{and}\;E_{i}{|}_{\mathrm{opt}}=-P^{\prime }(x_{\mathrm{tot}})x_{i}^{2},$$

with *x*_tot_ being determined by averaging the first equation over all agents,

2.22 $$P(x_{\mathrm{tot}})-\bar{c}=-\frac{x_{\mathrm{tot}}}{N}P^{\prime }(x_{\mathrm{tot}}).$$

The maximal per-unit cost *c*_max_ follows from the limit *x*_*i*_ → 0 in (2.21),

2.23 $$c_{\max}=P(x_{\mathrm{tot}})\;\text{and}\;x_{i}=\frac{c_{\max}-c_{i}}{-P^{\prime }(x_{\mathrm{tot}})},$$

which leads to

2.24 $$E_{i}{|}_{\mathrm{opt}}=\frac{{\left(c_{\max}-c_{i}\right)}^{2}}{-P^{\prime }(x_{\mathrm{tot}})}.$$

The dispersion relation is again strictly quadratic. It now depends explicitly on *x*_tot_, in contrast to the original case, see (2.9), which is, however, a minor alternation. Furthermore it follows from (2.22) that $c_{\max}-\bar{c}∼1/N$, the second precondition for catastrophic poverty. Catastrophic poverty arises hence for all monotonically decaying productivity functions *P*(*x*_tot_).

In (2.20), the individual investment costs are strictly linear in *x*_*i*_. Further on we will relax this assumption and discuss in detail the extension of the basic model to convex and concave cost functions. Another possible extension of (2.1) regards the value a certain amount of extracted resources has for the individual investor. This value may differ by a factor *r*_*i*_ between agents. The corresponding model,

2.25 $$E_{i}=x_{i}(r_{i}P(x_{\mathrm{tot}})-c_{i})=x_{i}r_{i}\left(P(x_{\mathrm{tot}})-\frac{c_{i}}{r_{i}}\right),$$

is functionally equivalent to (2.20). It then follows that the dispersion relation, which can be derived now for ${\tilde{E}}_{i}=E_{i}/r_{i}$, is still quadratic, albeit not as a function of *c*_*i*_, but of $\tilde{c}=c_{i}/r_{i}$.

### 2.9. Runaway exploitation

Our analysis is based on the condition that the level of the total investment does not diverge in the Nash equilibrium. This is indeed the case whenever $\bar{c}$ and *N* are finite, namely when $\bar{c}>0$ and *N* < ∞. For the original productivity function, *P* = exp (− *x*_tot_), total investment remains finite even in the limit $\bar{c}\to 0$, for which we found from (2.5) that *x*_tot_ → *N*. It can however happen, for other *P*(*x*_tot_), that the optimality condition (2.22) has no solution when $\bar{c}\to 0$. As an example we consider the productivity function

2.26 $$P(x_{\mathrm{tot}})=\frac{1}{{\left(1+x_{\mathrm{tot}}\right)}^{\beta }},$$

which decays as a power law for large total investments. The respective payoff function has a well-defined maximum for *N* = 1 and $\bar{c}=0$ when *β* > 1. For general values of *N*, the optimality condition (2.22) reduces to

2.27 $$x_{\mathrm{tot}}{|}_{\mathrm{opt}}\;\overset{\bar{c}\to 0}{=}\;\frac{N}{\beta -N}$$

when $\bar{c}\to 0$. The optimal total investment diverges hence even for finite numbers of agents, namely whenever *N* ≥ *β*. Instead, taking first the limit *N* → ∞ leads to $x_{\mathrm{tot}}={\left(1/\bar{c}\right)}^{1/\beta }-1$, which is positive and finite for all $0<\bar{c}<1$. Runaway exploitation can hence occur for finite *N*, but only when average per-unit costs are formally zero.^3^

One could characterize a given environment as resilient when its productivity decays only slowly with increased exploitation efforts. An example would be a fishing ground with robust regrowth rates. It is interesting in this regard that exponential functions decay faster with increasing argument than a power law. The possible occurrence of runaway exploitation for power law productivity functions suggests hence the hypothesis that the likelihood that existing environments will suffer from over-exploitation may raise with resilience. This conclusion is consistent with the results from a study of ecosystem exploitation that includes corruption traits [27].

### 2.10. Finite-size commons

Hitherto, investments of any size would result in positive nominal returns. Alternatively one can consider a common-pool resource for which there is an upper bound for total investments, *x*_max_. At this point, the productivity of the commons vanishes altogether. For simplicity we denote *x*_max_ as the size of the commons [22]. A possible productivity function is

2.28 $$P(x_{\mathrm{tot}})=1-\frac{x_{\mathrm{tot}}}{x_{\max}},$$

which leads via (2.22) to the optimal total investment

2.29 $$x_{\mathrm{tot}}{|}_{\mathrm{otp}}=(1-\bar{c})\frac{N}{N+1}x_{\max}.$$

This expression is functionally well behaved in the sense that runaway exploitation is absent for all parameter regimes, in contrast to (2.27). The optimal overall investment approaches the carrying capacity *x*_max_ of the commons when $\bar{c}\to 0$ and *N* → ∞. The dispersion relation (2.24) holds with *P*′(*x*_tot_) = −1/*x*_max_, which implies that catastrophic poverty is present. Catastrophic poverty will hence emerge for both finite-size and formally unbounded commons. We note that (2.28) was used by Ostrom for a laboratory protocol [22], as discussed later on in further detail.

## 3. Simulations

We solved the self-consistency condition (2.5) for *x*_tot_ numerically, which allows us to determine *c*_max_ = exp (− *x*_tot_) and individual investments *x*_*i*_, the latter from the linear relation (2.4). As expected, one finds perfect agreement between the direct evaluation of the payoffs, via *E*_*i*_ = (*c*_max_ − *c*_*i*_)*x*_*i*_, and the prediction of the dispersion relation (2.9).

**Figure 3.**
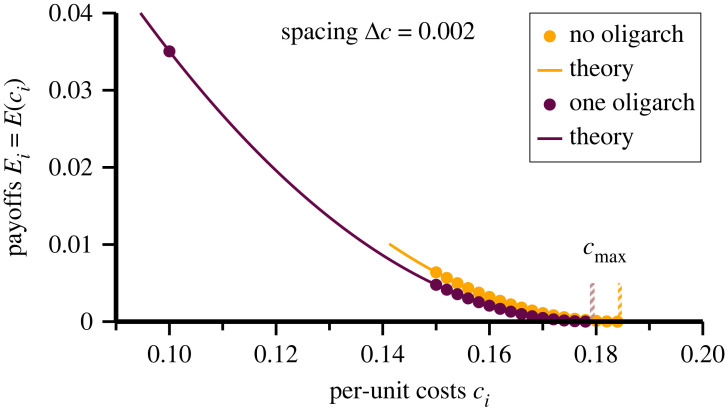
Dispersion relations. For a spacing of Δ*c* = 0.002 of consecutive per-unit costs, the payoffs *E*_*i*_ obtained from numerical simulations (filled circles). Given in comparison are the analytic results (lines), see (2.9). *Orange:* the payoffs for the surviving *N* = 18 agents, with $\bar{c}=0.167$ and *c*_max_ = 0.184 (striped vertical orange bar). Per-unit costs start at *c*_min_ = 0.15. *Brown:* one additional agent, the oligarch, is added at *c*_*i*_ = 0.1. One has now $\bar{c}=0.16$, *c*_max_ = 0.179 (striped vertical brown bar) and *N* = 16 total surviving agents (15 with *c*_*i*_ ≥ 0.15 plus the oligarch).

Particularly numerically accessible is the initial decimation procedure. Starting with a given distribution {*c*_*i*_} for the per-unit costs, one performs the decimation process iteratively until the number of surviving agents does not change any more. We concentrate here on distributions {*c*_*i*_} for which the bulk of the per-unit costs are uniformly spaced, *c*_*i*_ = *c*_min_ + *i*Δ*c*, compare figure 3. To the set of agents with equally spaced per-unit costs we added at most an additional agent, the oligarch, with a per-unit cost well below *c*_min_.

For the simulation presented in figure 3 we started with *N*_start_ agents. Without an oligarch, *N* = 18 agents remain after decimation when using *c*_min_ = 0.15 and Δ*c* = 0.002. The exact number of starting agents is irrelevant, as long as *N*_start_ ≥ 18, as agents with *c*_*i*_ > *c*_max_ are decimated out in any case. Lower numbers of *N*_start_ would lead in contrast to a different Nash state. For example, no decimation is performed when starting with a single agent.

### 3.1. No oligarchs

We first examine the results when there is no oligarch present. All calculations are for the reference model, as defined by (2.1), and for two final configurations, *N* = 18 and *N* = 1 (obtained by starting, respectively, with *N*_start_ ≥ 18 and *N*_start_ = 1). The data are given in table 1. We recall that the productivity of the commons,

3.1 $$P=e^{-x_{\mathrm{tot}}}=c_{\max},$$

coincides with the maximum per-unit cost, which is given in table 1. Alternatively, one can regard *P* = *c*_max_ as a measure for the degree of degradation, *viz.* for the status of the common resource.

**Table 1.** Simulation results. For regularly spaced per-unit costs, with *c*_min_ = 0.15 and Δ*c* = 0.002, the properties of the stationary state resulting from the initial decimation procedure. Compare figure 3. Given is the number *N* of surviving agents, total investment and payoff, *x*_tot_ and *E*_tot_, together with the average and maximal per-unit costs $\bar{c}$ and *c*_max_. The last two columns indicate if an oligarch at *c*_*i*_ = 0.1 was included and whether agents did cooperate.

*N*	*x*_tot_	*E*_tot_	$\bar{c}$	*c*_max_	oligarch	coop.
18	1.691	0.040	0.167	0.184	no	no
1	0.698	0.243	0.150	0.497	no	no
18	0.673	0.231	0.167	0.510	no	yes
15 + 1	1.720	0.061	0.160	0.179	yes	no
1 + 1	1.184	0.219	0.125	0.306	yes	no
15 + 1	0.683	0.236	0.160	0.505	yes	yes

Comparing the results for *N* = 18 and *N* = 1 one notes that the respective $\bar{c}$ differ by only about 10%. The total payoff *E*_tot_ is, however, reduced by a factor 6 ≈ 0.243/0.04 when increasing the population from *N* = 1 to *N* = 18. This reduction can be seen as a precursor of catastrophic poverty, as expressed by equation (2.11). For a given *c*_min_ the number of surviving agents depends on the spacing Δ*c*, with *N* increasing when Δ*c* is decreased. We tested that the (1/*N*)^2^ scaling characterizing catastrophic poverty on an individual level does indeed hold when *N* is further increased.

The numerical results show that overall welfare suffers substantially already for comparatively modest numbers of independently exploiting actors, here *N* = 18. An increased population size leads also to a deteriorating state of the commons, but to a somewhat lesser extent. The ratio of the productivity function *P* = *c*_max_ for *N* = 1, and, respectively, for *N* = 18, is 0.497/0.184 = 2.7, compare (3.1), which is about half the respective payoff ratio, 0.243/0.04 = 6.1. This effect will become less important for further increased population sizes, as *c*_max_ is bounded from below by $\bar{c}$.

Also included in table 1 is the data for *N* = 18 cooperating agents, using the basic protocol discussed further above, which is defined by *x*_*i*_ ≡ *x*_tot_/*N*. By construction, the mean $\bar{c}$ does not depend on whether agents do or do not cooperate. When cooperating, *E*_tot_ is substantially higher with respect to the same number on non-cooperating agents. The total payoff of cooperating agents is identical to that of a single agent with $c_{i}=\bar{c}$, but a bit reduced with respect to the case *N* = 1 shown in table 1, which is for a single agent with *c*_*i*_ = *c*_min_. In any case, cooperating agents do not suffer from catastrophic poverty.

### 3.2. A single oligarch

We added an additional agent with *c*_*i*_ = 0.1 to the setup described before. Without cooperation *N* = 16 = 15 + 1 agents survive decimation, the oligarch plus 15 bulk investors with *c*_*i*_ ≥ *c*_min_ = 0.15. The data are included in table 1 and shown in figure 3. Nominally, the total payoff is larger when an oligarch is present. Calculating the profits of the 15 bulk agents one finds a cumulative payoff of 0.0267, which is less than the payoff of the oligarch, 0.035. Overall, the addition of a single oligarch has only an indirect influence on the welfare of the remainder agents.

### 3.3. Participation window

**Figure 4.**
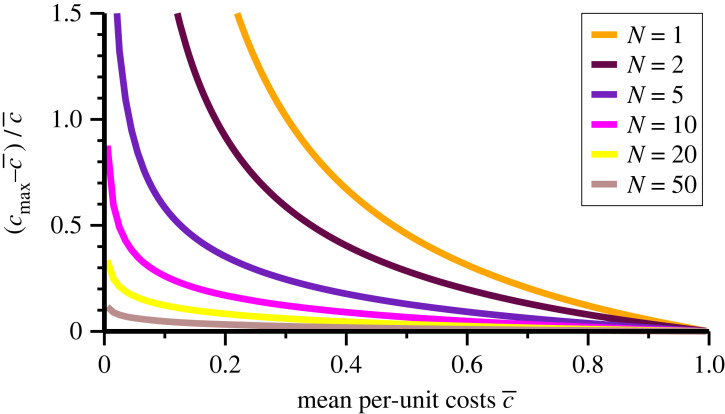
Window of participation. As determined by (2.6), the relative distance between maximal and mean per-unit costs, $\delta c_{p}=(c_{\max}-\bar{c})/\bar{c}$. The data are for fixed numbers *N* of agents. E.g. adding an agent to the case *N* = 1 will shift the curve to *N* = 2. The large-*N* limiting behaviour is $\log(1/\bar{c})/N$. For fixed *N*, the data diverge for vanishing mean per-unit costs. The window of participation for less efficient agents, *δc*_*p*_, can be interpreted also in terms of relative profit margins, see equation (3.3).

The per-unit cost of less efficient agents is located typically in the interval $\left[\bar{c},c_{\max}\right]$, which can hence be regarded as representing a window of participation. It is of interest to investigate how large this region is in relative terms. In figure 4, the results for

3.2 $$\delta c_{p}=\frac{c_{\max}-\bar{c}}{\bar{c}}=\frac{x_{\mathrm{tot}}}{N-x_{\mathrm{tot}}},$$

are shown, where we used (2.10) for *c*_max_. Note that $\lim_{\bar{c}\to 0}x_{\mathrm{tot}}=N$ holds, as shown in figure 2, which implies that the relative participation window *δc*_*p*_ diverges for $\bar{c}\to 0$. In the large-*N* limit one has $x_{\mathrm{tot}}\to \log(1/\bar{c})$ for $\bar{c}>0$ and hence $\delta c_{\;p}\to \log(1/\bar{c})/N$ when costs are small but finite.

For fixed population sizes *N* the curves for *δc*_*p*_ presented in figure 4 increase monotonically with decreasing average investment costs. Interestingly, one can regard $\bar{c}$ as a measure for the mean technological level of the investing group of agents. In this view, small average costs correspond to a technological advanced society, which is characterized by a large total investment *x*_tot_ and a correspondingly degraded commons. In addition, technologically advanced societies offer a substantial window of participation for less efficient agents, as expressed by a comparatively large $\delta c_{p}=(c_{\max}-\bar{c})/\bar{c}$, which implies that a substantial range of agents may survive the decimation process. This implies vice-versa that catastrophic poverty is less likely to arise in societies with large average costs $\bar{c}$.

The data for the window of participation presented in figure 4 can be interpreted in terms of relative profit margins *M*(*c*_*i*_). In the Nash state, the payoff per investment is

3.3 $$M(c_{i})=\frac{E_{i}}{c_{i}x_{i}}=\frac{c_{\max}-c_{i}}{c_{i}},$$

which follows directly from *E*_*i*_ = *x*_*i*_(*c*_max_ − *c*_*i*_). For a typical investor, with $c_{i}=\bar{c}$, the relative profit margin $M(\bar{c})$ coincides with *δc*_*p*_, see equation (3.2). For example, for *N* = 50 one has *δc*_*p*_ ≈ 0.12 for small $\bar{c}$, which corresponds to a typical profit margin of about 12%, still a healthy value. In the opposite limit, for small *N*, profit margins readily surpass 100% for efficient investors.

These considerations suggest two conclusions. First, that small populations will strongly attract additional investors in technologically advanced societies, namely when average costs are small. Second, efficient investors will make healthy profits even when *N* is substantial, say *N* = 50 or larger. At first sight, this observation seems to rule out the occurrence of catastrophic poverty. This is, however, not the case, as both $c_{\max}-\bar{c}$ and *x*_*i*_ scale with 1/*N* for per-unit costs within the window of participation, or close to $\bar{c}$ in general.

## 4. Convex and concave cost functions

The exploitation of a common resource by a group of self-centred agents can be described by a large class of models. Our discussions centred so far mostly on a specific implementation, the basic model (2.1), which we extended with regard to general productivity functions *P*(*x*_tot_), see (2.20). We examine now the effect of nonlinear cost functions,

4.1 $$E_{i}=x_{i}P(x_{\mathrm{tot}})-c_{i}C_{i}(x_{i})\;\text{with}\;C_{i}(0)=0\;\text{and}\;C_{i}^{\prime }(0)=1,$$

where we did write the cumulative individual investment costs as *c*_*i*_*C*_*i*_(*x*_*i*_). The parameterization used reduces to $c_{i}C_{i}(x_{i})=c_{i}x_{i}+O(x_{i}^{2})$ for small investments *x*_*i*_, with the prefactor *c*_*i*_ corresponding to the initial marginal costs [28]. The occurrence of catastrophic poverty depends on the functional form of the dispersion relation close to the profitability limit, *viz.* what happens when the *x*_*i*_ are small, which is governed in turn by the value of *c*_*i*_. On a functional level, *C*_*i*_(*x*_*i*_) and *c*_*i*_ correspond to a dimensionless cost function and the respective monetary scale.

### 4.1. Catastrophic poverty versus entry barriers

Catastrophic poverty will be present when the cost function is weakly concave, but not for strongly concave cost functions. This can be seen by considering the low-*x*_*i*_ expansion of the dimensionless cost function,

4.2 $$C_{i}(x_{i})=x_{i}-\gamma_{i}x_{i}^{2}+O(x_{i}^{3})\;\text{and}\;\gamma_{i}=\frac{-C_{i}^{\prime \prime }}{2}.$$

The dimensionless marginal costs decrease as *C*_*i*_′ ≈ 1 − 2*γx*_*i*_ when *γ*_*i*_ > 0, which can lead to the well-known phenomenon of an entry barrier [29,30]. Potentially, when economies of scale are impactful, agents make profits for large, but not for small investments. It is then not viable for a new actor to enter the existing market configuration with an initial small investment. When and how this happens depends in addition on the productivity function of the commons. For a detailed discussion, in particular also in regard to the emergence of catastrophic poverty, we will examine a concrete model.

### 4.2. Concave cost functions

For concreteness we use

4.3 $$C_{i}(x_{i})=\frac{1}{\gamma_{i}}\log(1+\gamma_{i}x_{i})\;\text{and}\;C_{i}^{\prime }(x_{i})=\frac{1}{1+\gamma_{i}x_{i}}$$

for the dimensionless cost function. For *γ*_*i*_ → 0 the original model (2.1) with constant marginal costs is recovered. To order $x_{i}^{2}$, the Taylor series of (4.3) reduces to (4.2). In general, the cost function specified by (4.3) is convex/concave, respectively, for *γ* < 0 and *γ* > 0. Note that costs diverge logarithmically for *x*_*i*_ → 1/|*γ*| when *γ* < 0.

For a reference model with nonlinear costs we set *γ*_*i*_ ≡ *γ* and *P*(*x*_tot_) = exp(− *x*_tot_). The payoff functional is then

4.4 $$E_{i}=x_{i}e^{-x_{\mathrm{tot}}}-\frac{c_{i}}{\gamma }\log(1+\gamma x_{i}),$$

which leads to the gradient

4.5 $$\frac{dE_{i}}{dx_{i}}=(1-x_{i})e^{-x_{\mathrm{tot}}}-\frac{c_{i}}{1+\gamma x_{i}}.$$

The locus of the *x*_*i*_ = 0 crossing of the gradient *E*_*i*_′ = d*E*_*i*_/d*x*_*i*_ is given by exp(− *x*_tot_) = *c*_max_. It holds as before that *c*_max_ coincides with the profitability boundary for moderate values of *γ*. The situation changes, however, for larger values of *γ*, as shown further below. Setting *E*_*i*_′ = 0 yields

4.6 $$1+x_{i}(\gamma -1)-\gamma x_{i}^{2}=c_{i}e^{x_{\mathrm{tot}}}\;\text{and}\;x_{i}=\frac{\gamma -1}{2\gamma }\pm \sqrt{{\left(\frac{\gamma -1}{2\gamma }\right)}^{2}+\frac{c_{\max}-c_{i}}{\gamma c_{\max}}}.$$

The negative/positive root corresponds to the dynamical stable solution, respectively, for *γ* < 0 and *γ* > 0, which can be shown by a standard stability analysis. In both cases, the *γ* = 0 result *x*_*i*_ = (*c*_max_ − *c*_*i*_)/*c*_max_ is recovered in the limit *γ* → 0. Compare (2.4).

**Figure 5.**
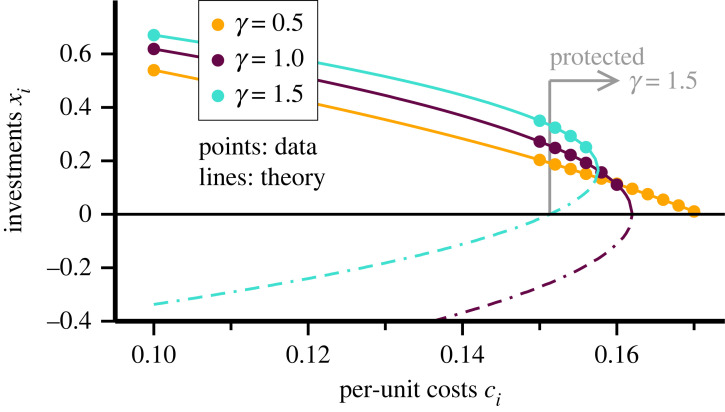
Protected exploitation. Results for a logarithmic cost function, as specified by equation (4.5), with *γ* ≥ 0 encoding how fast marginal costs fall when investments are increased. The data have been obtained by adapting the individual *x*_*i*_ along the gradient *E*_*i*_′ = d*E*_*i*_/d*x*_*i*_ until convergence (points). The solid line corresponds to the positive root of the stationarity condition (4.6). For *γ* = 1.0/1.5, the respective negative root is also shown (dash-dotted lines). For *γ* = 1.5, a dynamical entry barrier exists in the region indicated by the grey arrow, as explained further in figure 6. As for figure 3, cost multipliers *c*_*i*_ = 0.15 + *i*Δ*c* have been used, with Δ*c* = 0.002. In addition a single oligarch at *c*_*i*_ = 0.1 has been included. After decimation, *N* = 12/7/5 agents remain for *γ* = 0.5/1.0/1.5.

Results from numerical simulations together with (4.6) are presented in figure 5. For *γ* < 1 one finds that *x*_*i*_ crosses zero with a finite slope. This is equivalent to the linear behaviour observed for the original model, compare equation (2.4). All considerations regarding the occurrence of catastrophic poverty hence remain valid for convex and moderately concave cost functions, *viz.* for *γ* < 1. We call this region the CP phase (CP for catastrophic poverty).

### 4.3. Protected exploitation

By definition, *c*_max_ is the locus at which the gradient of the individual payoffs (4.5) changes sign when the individual investments are vanishingly small,

4.7 $$\frac{dE_{i}}{dx_{i}}{|}_{x_{i}=0}=c_{\max}-c_{i}.$$

As shown in figure 5, agents with a cost multiplier *c*_*i*_ > *c*_max_ may survive, nevertheless the decimation process when their individual investments *x*_*i*_ are substantial. This is, as discussed above, the telltale sign of an entry barrier. Existing agents are hence protected against new competitors when *c*_*i*_ > *c*_max_. There is, however, a limit. Regardless of the size of their investments, agents will not survive when cost factors become too large. This happens when the stationarity condition *E*_*i*_′ = 0 cannot be fulfilled any more.

We denote with *c*_node_ the point at which the two roots for *x*_*i*_ given by (4.6) merge. One finds

4.8 $$c_{\mathrm{node}}=c_{\max}+c_{\max}\frac{{\left(\gamma -1\right)}^{2}}{4\gamma }=c_{\max}\frac{{\left(\gamma +1\right)}^{2}}{4\gamma }.$$

No solution for *x*_*i*_ exists for *c*_*i*_ > *c*_node_, which marks hence the upper bound of the protected area. As expected, one has that *c*_node_ coincides with *c*_max_ for *γ* = 1 and that *c*_node_ diverges for *γ* → 0. In terms of dynamical systems theory, *c*_node_ corresponds to a saddle–node bifurcation [24].

### 4.4. Forced market exits

**Figure 6.**
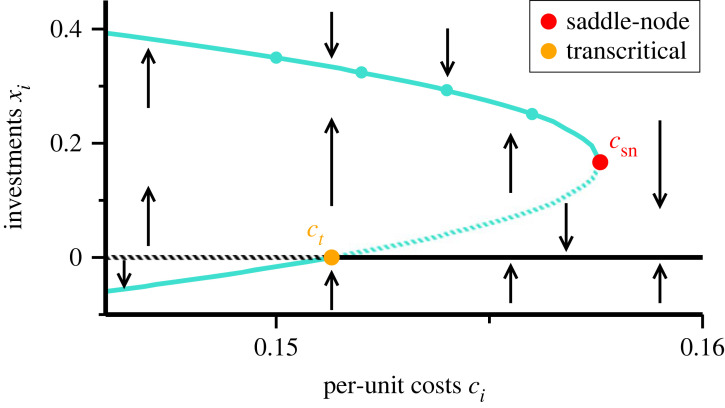
Frozen bifurcation diagram. An enlargement of figure 5, showing the flow for *γ* = 1.5 when *c*_max_ = exp(− *x*_tot_) is kept constant (frozen). The arrows indicate the direction of the gradients *E*_*i*_′ = d*E*_*i*_/d*x*_*i*_ of the payoffs (the flow, in dynamical systems terminology), as given by (4.5). Solid/shaded lines correspond to stable/unstable solutions of *E*_*i*_′ = 0. At the saddle–node bifurcation (red filled circle) occurring at *c*_node_, the stable and the unstable manifolds merge, corresponding, respectively, to the positive/negative roots defined in (4.6). The unstable manifold undergoes a transcritical transition (orange filled circle) at *c*_max_, when crossing the *x*_*i*_ = 0 line. For *c*_*i*_ > *c*_max_ the *x*_*i*_ = 0 line is stable, which means that there is a dynamical barrier for newcomers to enter the market.

In order to understand what happens beyond the protected area we present in figure 6 the frozen bifurcation diagram for *γ* = 1.5. It is given by the flow *E*_*i*_′ under the constraint that $x_{\mathrm{tot}}=\sum_{j}x_{j}$ is kept constant, *viz.* frozen. This approximation can be used to examine the stability of the nullkline *E*_*i*_′ = 0 in the vicinity of the Nash equilibrium. Further away, when individual investments *x*_*i*_ deviate substantially from their stationary value, the frozen approximation will, however, break down. Together with the saddle–node bifurcation at *c*_*i*_ = *c*_node_, one observes in figure 6 that the negative root of (4.6) crosses *x*_*i*_ = 0 at *c*_max_ via a transcritical bifurcation. The latter expresses that the nullkline *x*_*i*_ = 0 switches stability at *c*_*i*_ = *c*_max_, as given by (4.7).

We now examine what happens if a selection of efficient agents further improve their cost functions. We assume that this happens slowly, such that the system remains all the time close to the Nash equilibrium. One is then in a quasi-stationary state. At the same time the cost multiplier of the least efficient surviving agent, denoted here by *c*_*N*_, is assumed to be constant. Both *c*_max_ and *c*_node_ decrease when some agents lower their *c*_*i*_ progressively, here for *i* < *N*. Eventually, the least efficient agent will be squeezed out of the market. The dynamics of this forced market exit differs qualitatively for *γ* < 1 and *γ* > 1, *viz.* as a function of whether entry barriers are present.

For *γ* < 1 catastrophic poverty is present and the optimal investment *x*_*N*_ of the least efficient agent vanishes linearly, ∼(*c*_max_ − *c*_*N*_), when *c*_max_ decreases. The point of the market exit can hence be predicted by analysing the rate at which *x*_*N*_ deceases.

For *γ* > 1 the optimal investment *x*_*N*_ does not vanish when *c*_*N*_ approaches *c*_node_. Right at the saddle–node bifurcation the optimal investment *x*_node_ is finite, which can be seen by inserting (4.8) into (4.6). Analytically a square-root singularity exists, namely that $x_{N}-x_{\mathrm{node}}∼\sqrt{c_{\mathrm{node}}-c_{N}}$. Agents will, however, not be able to resolve this singular dependence under realistic market conditions involving substantial scattering and noise. The least efficient investor will hence not be able to predict its own market exit by observing *x*_*N*_. The forced market exit occurs in this sense by a sudden death.

At first sight, the picture developed here may seem inconsistent. If *x*_node_ is finite it follows that agents with *c*_*i*_ = *c*_node_ make small but finite profits, which can be verified by evaluating the payoff at the point of the bifurcation. There is hence no incentive for the least efficient agent to stop investing into the common resource. What happens is more subtle. The flow becomes negative when *c*_*N*_ exceeds *c*_node_ by any amount, as is evident from the frozen phase diagram presented in figure 6. The value of *x*_*N*_ decreases for any *c*_*N*_ > *c*_node_, which leads in turn to a reduced contribution of the least efficient investor to *x*_tot_. In response, the remainder agents will increase their own investments, which will further reduce *c*_max_, and consequently also the optimal *x*_*N*_. In the end, *x*_*N*_ will drop to zero.

Effectively, the sudden death forced market exit results from a collective reorganization of the market. The agents collectively adapt their optimal investment, which occurs successively. The timescale of the ‘sudden death’ forced exit is hence determined by the typical time needed to reach a new Nash equilibrium. The exit is ‘sudden’ only insofar that the agent cannot predict the starting point.

## 5. Discussion

Based on a concept proposed by William Forster Lloyd in the nineteenth century, Garrett Hardin examined in 1968 the impact of increased exploitation pressures on the state of global natural resources [31]. An exploration of a specific mathematical modelling framework for the possible outcome, the TOC, was, however, not performed at this point. At the centre of attention for the TOC are common-pool resources for which access is unrestricted and free of charges. However, free access does not imply that utilization will not entail monetary costs. E.g. for Lloyd’s classical example, an open-to-all pasture of a village, peasants need to invest first in their respective livestocks. One needs animals, say cows, in order to benefit from a common grazing ground.

For an experimental setup, Ostrom [22] used a model functionally identical to (4.1). Agents are assumed to dispose of an asset that can be invested either into a default activity, like working for an employer and getting paid, or into a common pool of resources. Assets not used for the default activity correspond to opportunity costs [23], which implies that they are functionally equivalent to investment costs. It is hence reasonable to assume, as done throughout this paper, that agents need to invest when accessing a common pool of resources. An equivalent observation holds for certain classes of managed commons. An example would be the case of a taxed fishing ground. The incurring costs would be equivalent to investment costs if taxes are proportional to the capacity of the individual boats, but otherwise independent of the actual size of the catch.

Lloyd & Hardin suggested that ‘the more the better’ would be the optimal strategy for the independently acting agent whenever access to resources is not restricted, *viz.* when the commons are left unmanaged [31]. If at all, this can, however, be the case only when investment costs are non-existent: the individual agent will stop increasing the size of its investment at least once the productivity of the commons falls below its own per-unit investment costs. Exploitation rates do not need to diverge, however, even when investment costs are negligibly small. We showed that large-scale extraction of a common resource will be limited when the productivity of the commons degrades exponentially with total investment *x*_tot_. This statement holds whenever the number *N* of participating agents remains finite, *viz.* when *N* < ∞. However, runaway exploitation occurs for vanishing investment costs when the environment degrades not exponentially, but as a power law, see (2.27). This case could hence be considered, in the sense of Lloyd & Hardin, to correspond to the paradigmal TOC scenario.

A particular focus of our investigations regards the dependence of the TOC Nash equilibrium on group size, *viz.* on *N*. A consistent finding is that smaller groups will enjoy, ceteris paribus, larger average payoffs. In the extreme case of runaway exploitation, as defined by (2.27), payoffs drop to zero when the group size exceeds a certain threshold, remaining finite below.

The effect of group size has been studied intensively in game-theoretical studies [32], in particular with regard to the emergence of collaboration [33]. Also of relevance in this context are psychological and moral components [34–36], like trust and the decoy effect [37,38], longer-term perspectives [39], collective and feedback effects [40–43], and evolutionary drives [11,44]. A main focus of these and other studies of the TOC regards the circumstances under which over-exploitation will be avoided. Possible solutions include property management [45], enhanced social reputation [46], buffer zones [47], social diversity [48] or community-based institutions in general [7]. For a general analysis see [49].

Here, we proposed an alternative view, pointing out that the steady-state of the pure TOC problem is highly non-trivial already by itself. Commons with unrestricted access are subject to only modest levels of exploitation when group sizes are small, and/or when investment costs are substantial. The problem becomes severe, however, when the investment costs of a large number of self-centred investors are small, which can be expected to be the case for technologically advanced societies. The majority of agents will necessarily suffer from substantially reduced incomes whenever group sizes are large, scaling as (1/*N*)^2^. This phenomenon is called ‘catastrophic poverty’.

Exploitation does not need to be massive for catastrophic poverty to emerge, which implies that the status of the commons is not the only determinant. The steady-state size of the total investment *x*_tot_ may be high, but whether this value would be sustainable for a specific real-world pool of resources, or not, this question transcends the modelling framework used here. Instead, agents increase their investments until the majority of the population is close to a self-consistently determined profitability threshold, with the distance to the profitability threshold (*viz.* the subsistence level) scaling as 1/*N* in equilibrium. A limited number of agents with reduced investment costs, the oligarchs, may be present in addition. Oligarchs escape catastrophic poverty.

Previous studies focused mostly, but not exclusively [48], on agents with identical characteristics [2]. The variability between the individual actors played then a non-essential role. Catastrophic poverty will occur also in this limit, *viz.* for the case of identical agents. The dependency of the Nash equilibrium on the differences between the individual agents is nevertheless an integral part of our investigations. To be specific, agents have individual per-unit investment costs *c*_*i*_. The functional relation between *c*_*i*_ and the steady-state individual payoff, *E*(*c*_*i*_), is key for the understanding of catastrophic poverty. We showed that this relation, the dispersion relation, is strictly quadratic, *E*(*c*_*i*_) = (*c*_max_ − *c*_*i*_)^2^/*c*_max_, where *c*_max_ is the profitability threshold. Profits vanish hence quadratically close *c*_max_, and not linearly, which is the cause of catastrophic poverty.

Agents will exploit a common pool of resources only when making a net gain. An intriguing finding is that the expected number of actively harvesting agents increases with decreasing investment costs. This implies that a given resource will be exploited more severely when improved technologies are introduced. This observation may explain in reverse why self-organized governance of the commons is regularly found in communities with somewhat limited access to modern technologies [50].

Our findings hold for a large class of TOC-models. Of minor relevance for the occurrence of catastrophic poverty is the specific dependency of the productivity of the commons on total investment. Regarding the structure of investments costs, catastrophic poverty is present for convex and moderately concave cost functions. A collective reorganization of the market, however, takes place for strongly concave cost functions. In this phase, catastrophic poverty is absent. Instead, one finds that entry barriers for prospective new investors emerge and that the dynamics of forced market exits changes on a qualitative level.

## Data Availability

This article has no additional data.

## Footnotes

1 In physics terms [24,25], the cumulative investment acts as a mediating molecular field.

2 In physics, the dispersion relation describes the functional dependency of the frequency (or energy) of an excitation with respect to the controlling variable, in general, the wavevector [26]. In analogy, equation (2.9) specifies the relation of the payoff *E*_*i*_ (taken to correspond to the energy) to the controlling degree of freedom, in this case, the per-unit cost *c*_*i*_.

3 In the real world, costs can be small, but they are never exactly zero. The limit $\bar{c}\to 0$ corresponds in this sense to a formal consideration.
